# Case Report: Tocilizumab in pediatric febrile infection-related epilepsy syndrome

**DOI:** 10.3389/fimmu.2026.1767388

**Published:** 2026-06-03

**Authors:** Lingyu Pang, Xuefang Liu, Suzhen Sun, Fang Chen, Hongru Lu, Yakun Du, Xin Li

**Affiliations:** 1 First Department of Neurology, Hebei Children’s Hospital, Shijiazhuang, China; 2 First Department of Neurology, Hebei Provincial Clinical Research Center for Child Health and Disease, Shijiazhuang, China; 3 First Department of Neurology, Hebei Provincial Key Laboratory for Pediatric Epilepsy and Neurological Disorders, Shijiazhuang, China

**Keywords:** child, febrile infection-related epilepsy syndrome, IL-6, immunotherapy, tocilizumab

## Abstract

**Background and purpose:**

We describe the clinical characteristics and the therapeutic effect of tocilizumab with Febrile Infection-Related Epilepsy Syndrome (FIRES). FIRES is characterized by super refractory status epilepticus (SRSE) in previously healthy children, following a febrile illness that manifests between 2 weeks and 24 hours prior to the onset of seizure.

**Methods:**

We collected and summarized the clinical data of two pediatric patients with FIRES and compare with those described previously in the literature.

**Results:**

Elevated levels of IL-6 were observed in the two patient’s blood samples. Patient 1 consented to receive tocilizumab on the 58th day of the disease progression, resulting in transient relief of seizures. Throughout the follow-up period, the patient remained unconscious and consistently experienced intermittent seizures. Patient 2 was administered tocilizumab on the 7th day of the disease course. During the six-month follow-up, no seizures were observed and no significant cognitive decline. Up to date, 7 patients of pediatric FIRES treated with tocilizumab have been reported. The clinical symptoms of the patients were relatively consistent, characterized by focal seizures and status epilepticus as the primary manifestations. Serum IL-6 may exhibit elevated or normal, whereas cerebrospinal fluid IL-6 demonstrated a significant elevation, indicating a lack of concordance.

**Conclusion:**

Now little has been known about the administration of tocilizumab in pediatric FIRES. We posit that early administration of tocilizumab (within 3 weeks from disease onset) positively impacts the treatment of FIRES.

## 1 Introduction

New-onset refractory status epilepticus (NORSE) is a rare and devastating condition (1). The febrile infection-related epilepsy syndrome (FIRES) was redefined as a subgroup of NORSE preceded by a febrile illness between 2 weeks and 24 hours before the onset of refractory status epilepticus (2). It appears in patients without active epilepsy or relevant previous neurological diseases, without a clear acute or active structural, toxic, or metabolic cause (3). Global brain atrophy often occurs within a few weeks and is usually associated with a poor outcome (4). FIRES can be caused by autoimmune encephalitis, uncommon central nervous system infections and genetic disorders, but most cases remain cryptogenic (1).

Recent clinical and basic research findings strongly suggest that cryptogenic cases are caused by a postinfectious auto-inflammatory mechanism involving the innate immunity system (1). The management of cryptogenic FIRES using antiseizure medications, anesthetic drugs, and conventional immune therapies often yields unsatisfactory results and is accompanied by adverse effects (5). Therapeutic strategies targeting innate immunity pathways, such as inhibitors of interleukin-1β and interleukin-6 pathways, hold great promise in the field. Tocilizumab, an inhibitor of the IL-6 receptor (IL-6R), blocks IL-6 mediated signal transduction, which has been proven to enhance the outcome of some refractory autoimmune neurologic diseases, and its efficacy has also been evaluated in acute refractory seizures (6). However, data remain very scarce in children. Herein, we present two new cases of FIRES treated with tocilizumab, to enrich the treatment experience of FIRES and provide a basis for precise targeted therapy.

## 2 Patients and methods

### 2.1 Patients

This study retrospectively analyzed two pediatric patients diagnosed with febrile infection-related epilepsy syndrome (FIRES) who were admitted to the children hospitalized at Hebei Children’s Hospital between Augest and October 2023.

### 2.2 Data collection and literature review

Clinical data were retrospectively collected from medical records, including clinical manifestation, medical and family history, laboratory findings, neuroimaging and electroencephalographic results, treatment strategies, and clinical outcomes.

We conducted a systematic literature search (between January 1930 and December 2025) from PubMed using different combination of search words as follows:”NORSE,” “FIRES,” “new-onset refractory status epilepticus,” “febrile infection-related epilepsy syndrome,” “refractory status epilepticus,” and “super-refractory status epilepticus”. To maintain consistency, we specifically focused on articles published in the English language. In an attempt to ensure all data were appropriately extracted, four authors reviewed and assessed the results.

## 3 Results

### 3.1 Case presentation

#### 3.1.1 Case 1

**Figure 1:**
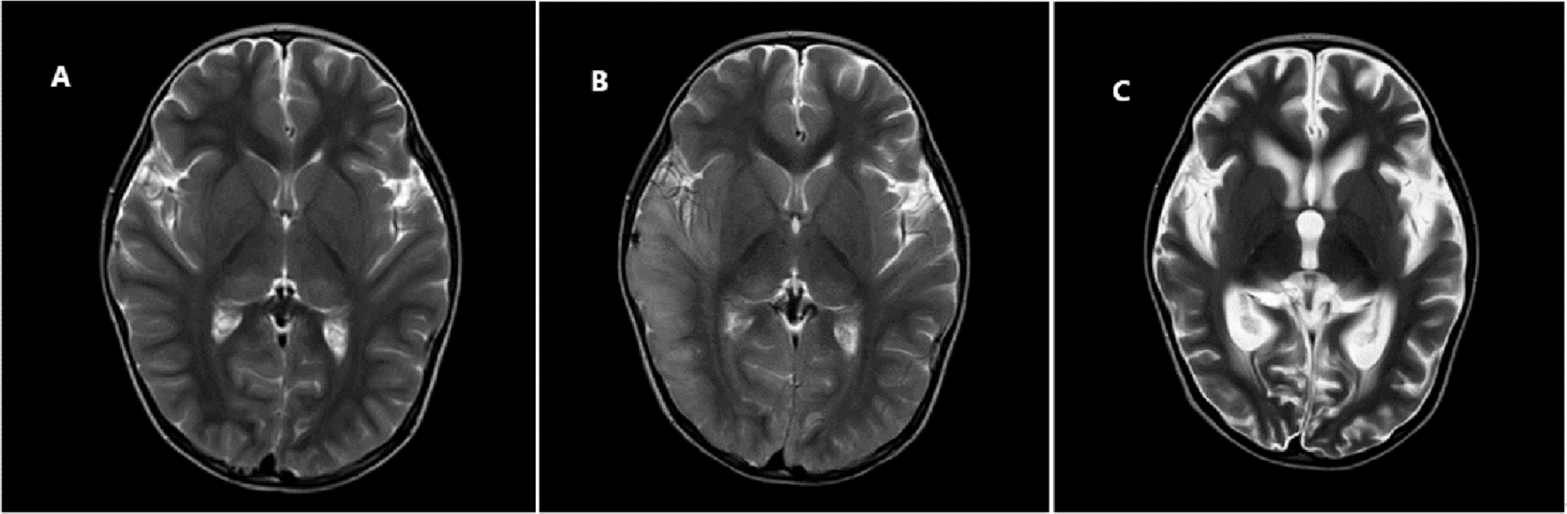
Serial MRIs on days 2 **(A)** and 16 **(B)** showed progressive abnormal signals in the bilateral thalamus, right occipital lobe and multiple cortices; on day 93 **(C)** was generalized brain atrophy.

A healthy 34-month-old female child presented with four episodes of loss of consciousness, persisting fixed gaze and cyanosis lasting between 2 minutes and half an hour. She had a fever for 4 days before the episodes, which lasted for 2 days and became asymptomatic afterward. Phenobarbital and diazepam were given initially, but seizures increased in frequency along with a high fever, necessitating admission to the Pediatric Intensive Care Unit (PICU). Family history was irrelevant for specific neurologic disorders and previous neurologic manifestations of the child were denied. Soon after the admission the child experienced frequent focal seizures and underwent treatment with intravenous midazolam (0.1mg/kg/hours) in an attempt to alleviate the seizures, but the seizures were persistent. Video-electroencephalogram (VEEG) revealed diffuse medium-high amplitude slow waves ranging from 1.0 to 2.5Hz, along with dispersed spike waves and spike slow waves in the right anterior middle temporal region. The cerebrospinal fluid (CSF) analysis revealed normal findings (biology, virology, bacteriology). Metabolic and neuronal antibody screening was negative in blood and CSF samples. The cranial magnetic resonance imaging (MRI) showed abnormal signals in the bilateral thalamus, right occipital lobe and part of the cortex (**Figure 1A**). Given the abrupt clinical onset, a possible autoimmune etiology was assessed. Intravenous immunomodulator treatment, immunoglobulins and corticosteroids, were associated without improvement. On the 16th day of the disease progression, VEEG demonstrated frequent focal discharges and epileptic status, concomitant with a serum interleukin-6 level of 14.39 pg/ml. Subsequent head MRI revealed multiple abnormal signals (**Figure 1B**).

**Figure 2:**
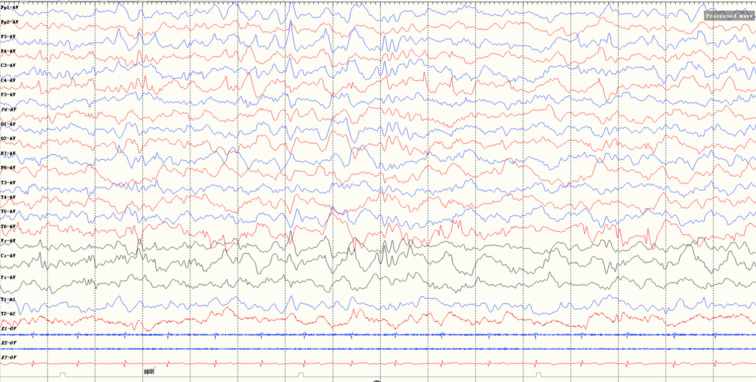
VEEG revealed diffuse 2-3Hz δ-slow wave, multifocal sharp waves, sharp slow waves during sleep; NCSE.

Despite multiple attempts with various combinations of anti-seizure medications (ASMs) including topiramate (TPM), perampanel (PER), oxcarbazepine (OXC), clonazepam (CZP), lacosamide (LCM), and implementing continuous intravenous perfusion (CIP) of midazolam, amobarbital along with the ketogenic diet (on day 15 of the course), the epileptic state ceased on day 38. However, consciousness did not recover. Subsequent video EEG revealed persistent diffuse slow wave activity evolving to non-convulsive status epilepticus (NCSE) (**Figure 2**). The family initially declined the recommendation to administer tocilizumab. The interleukin-6 level of 84.17 pg/ml (reference <20pg/ml) was reassessed on the 54th day of the disease course, and the family consented to administer tocilizumab this time. Pre-administration examination was conducted, followed by administration of 4 doses of tocilizumab (12mg/kg) starting from day 58, at intervals of every 2-4 weeks for each dose. After the second dose of tocilizumab was applied, the convulsions were gradually controlled, the VEEG indicated no NCSE, and the consciousness was improved than before, accompanied by a decrease in interleukin-6 levels measuring at 38.66 pg/ml.

On day 89 of the disease course, a urinary tract infection occurred with an elevated interleukin-6 level of 134.16 pg/ml upon re-examination. Recurrent seizures occurred on the 105th day of the illness course, while VEEG reassessment demonstrated focal seizures in the frontal region, concomitant with the reappearance of NCSE. Furthermore, brain atrophy is evident on head MR I (**Figure 1C**). The administration of immunoglobulin was repeated, and the fourth dose of tocilizumab was administered on day 109. The level of interleukin-6 decreased to 43.86 pg/ml. The convulsive episodes in the child exhibited a reduction compared to previous occurrences, with no apparent seizures observed for a maximum duration of approximately 20 days, Interleukin-6 was measured at 1.54 pg/ml on day 145. However, the level of consciousness remained impaired.

#### 3.1.2 Case 2

**Figure 3:**
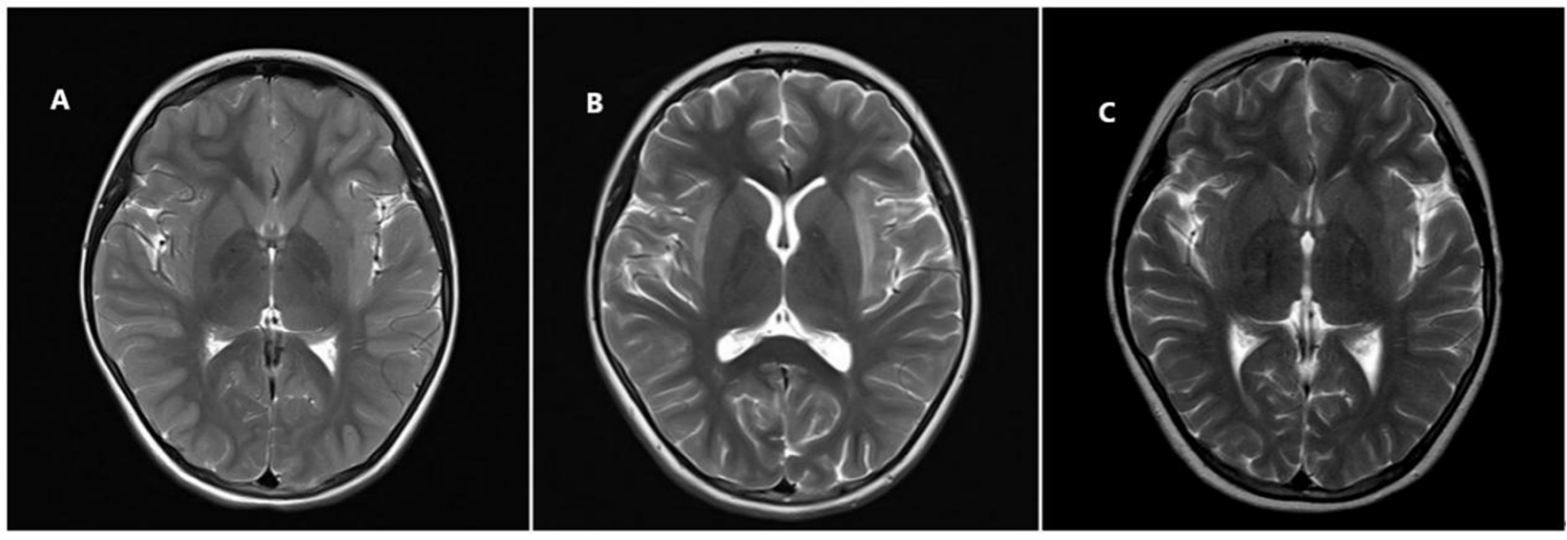
Serial MRIs on day 2 **(A)** was normal, and on day 9 **(B)** showed multiple abnormal signals with restricted diffusion in the bilateral frontoparietal occipital cortex, subcortical cortex, and bilateral insular lobe; finally, on day 42 **(C)** multiple aberrant signals had been absorbed and sulci were mildly widened.

A previously healthy 9-year-old girl initially presented with drowsiness, which was followed by nocturnal urinary incontinence. She then experienced an episode of seizure during her waking period, which was characterized by loss of consciousness, fixed gaze, cyanosis, and limb stiffness, lasting approximately five minutes. She had two episodes of fever 10 days before, each lasting three days, followed by asymptomatic conditions afterward. Family history is negative for neurologic disorders. After admission, the patient developed moderate fever again, and frequent focal seizures were observed on the second day of the disease course. Video-electroencephalogram revealed diffuse 1-2Hzδ slow waves during sleep, and a focal seizure originating from the right temporal region was recorded during sleep. Brain MRI showed no abnormal signal (**Figure 3A**). Cerebrospinal fluid analysis normal white blood cell count, glucose and protein. The metabolic, autoimmune, and neuronal autoantibody plasma and CSF screening was negative. CSF targeted next-generation sequencing (tNGS) was negative. Serum IL-6 level was 6.09pg/ml. Considering the presence of immune factors, the patient received immunoglobulin and high-dose methylprednisolone therapy, attempted multiple ASMs including LCM, clobazam (CLB), PER, phenobarbital (PB) in various combinations, and implemented CIP of midazolam. The patient continued to experience frequent seizures accompanied by a moderate to low-grade fever. Seizures were exacerbated during the onset of elevated body temperature, characterized by tremors in the left mouth and facial muscles, along with drooling. Confusion was observed during episodes of frequent seizures, and there was a possibility of tremors occurring in the left limb as well.

**Figure 4:**
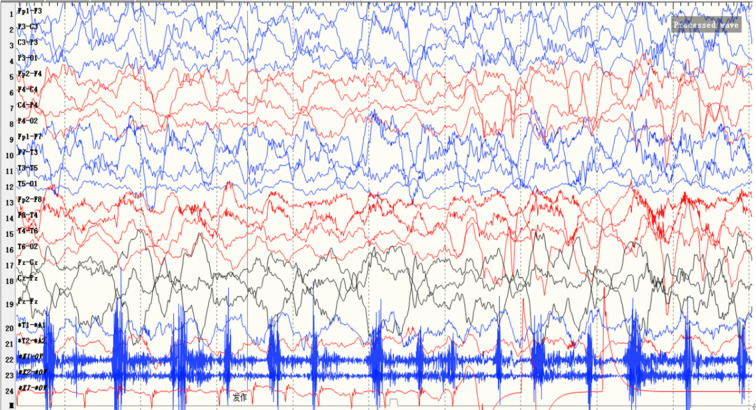
VEEG showed frequent focal seizures, EPC.

On the 5 days of illness progression, reexamination of the VEEG revealed frequent focal seizures, and epilepsia partialis continua (EPC) (**Figure 4**). Additionally, IL-6 reexamination yielded a result of 19.54pg/ml. FIRES was considered because first-line immunotherapy was ineffective. The patient’s condition was communicated with the family and tocilizumab was recommended. Family members agreed to use, and on the 7 days of the disease course, the first dose of tocilizumab (12mg/kg) was administered. On the 9th day of disease progression, cranial MRI demonstrated multiple abnormal signals with restricted diffusion in the bilateral frontoparietal occipital cortex, subcortical cortex, and bilateral insular lobe (**Figure 3B**). By the 19th day of illness onset, resolution of left angle mouth tremor was observed leading to a gradual discontinuation of midazolam while maintaining oral administration of ASMs (LCM, CLB, PER). Subsequently on day 21 following disease onset re-administration for a second dose of tocilizumab therapy alongside monitoring interleukin-6 (IL-6) levels which were quantified at 7.82 pg/ml. EEG recording performed 2 days after the second administration of tocilizumab revealed generalized slow wave activity predominantly localized in temporal regions. On the 42nd-day review, VEEG prompted slowly evolving background activities without epileptic waves. Additionally, the head MRI revealed multiple aberrant signals had been absorbed and sulci were mildly widened (**Figure 3C**). Furthermore, IL-6 was reviewed and found to be at 14.13 pg/ml. After half a year of follow-up, the patient had no seizures or cognitive impairment.

### 3.2 Literature review

To date, 7 pediatric FIRES patients treated with tocilizumab have been reported (7–11), including the two presented here. Their treatment history and prognosis are shown in **Table 1**.

**Table 1:** Treatment history and prognosis of children with febrile infection−related epilepsy syndrome treated with tocilizumab.

Patient No.			1	2	3	4	5	6	7
Clinical data	Gender		Male	Male	Male	Male	Female	Female	Male
	Age of onset		6Y	9Y	9Y	5Y	2.8Y	9Y	11Y
	Time from fever to seizure onset		7days	5 days	3 days	2 days	4 days	10 days	2days
	Seizure type		Focal seizures	Focal motor seizures	Focal seizures	Focal seizures	Focal seizures	Focal seizures	Focal seizures
	Duration of status epilepticus		23 days	21 days	18 days	39 days	38 days	18 days	7days
	CSF IL-6		Day 17 84pg/mL (≤25pg/mL)	*N/K*	31.4 pg/mL	149.9 pg/mL	*N/K*	*N/K*	50.4pg/mL
	Blood	Initial IL-6	Day 17 normal	Day 4 and day 17 normal	14 pg/mL (0-7 pg/mL)	Normal	Day 13 14.39pg/ml; day 54 84.17 pg/ml	Day2 6.09pg/ml, Day 5 19.54pg/ml	*N/K*
		IL-6 after tocilizumab administration	*N/K*	*N/K*	2.9 pg/mL (0-7 pg/mL)	Normal	Day 117 43.86 pg/ml	Day 43 14.13pg/ml	*N/K*
Treatment	Mechanical ventilation		*N/K*	Day 1 to day 19	*N/K*	*Y*	*Y*	*N*	*N*
	Immunotherapy		IVIG, IVMP, anakinra	IVIG, IVMP	*N*	IVIG, IVMP	IVIG, IVMP	IVIG, IVMP	IVIG, IVMP
	Plasma exchange		*N/K*	Six plasma exchanges from day 11 to day 16	Yes, from day 14	*N*	*N*	*N*	*N*
	ASMs		LEV, PB, VPA, LCM, TPM, CBD, LZP	CZP, PHT, LEV, PB, LCM	VPA, LEV, LCM, PHT, PB	PB, VPA, LEV, TPM, OXC, LCM, CZP, PER, ZNS	TPM, PER, OXC, CZP, LCM	LCM, CLB, PER, PB	LEV,VPA, CLB
	Anesthetics		Midazolam, ketamine, pentobarbital	Midazolam, propofol, ketamine	Midazolam, ketamine	Midazolam, propofol	Midazolam, amobarbital	Midazolam	N/K
	Ketogenic diet		Yes, from day 14	Yes	Yes	N/K	Yes, from day 15	*N*	Yes, from day 10
	Time points of tocilizumab Administration (since first convulsion)		Day 20, every 2 weeks,4 doses over 8 weeks	On day 17 and day 23	Day 21, every 1 week,2 doses	Day 36, every 1 week,2 doses	Day 58, every 2 weeks,4 doses over 8 weeks	Day 7, every 2 weeks, 2 dose	Day 10,Day 23,Day 55,Day 88
	Dose of tocilizumab		12mg/kg	4 mg/kg	4 mg/kg	4 mg/kg	12mg/kg	12mg/kg	8.8mg/kg
Therapeutic effect			Seizure burden decreased 3 days after the first administration of tocilizumab. Seizures did not recur with subsequent discontinuation of tocilizumab or ketogenic diet.	Seizures stopped 4 days after the first administration of tocilizumab. The child rapidly recovered his previous language level and motor abilities. A 24-h EEG recording performed on 4 days after the second administration of tocilizumab revealed normal background activity, without any seizures.	The patient improved globally, and the seizures began to decrease in frequency. VEEG 13 days after the second dose, the critical patterns had disappeared.	3 days after the first dose, the seizures were reduced by more than 50%. 10 days after the second dose, the seizures were controlled.	The seizures had a transient resolution, but the level of consciousness remained impaired.	Seizures stopped 12 days after the first administration of tocilizumab. The child rapidly recovered his previous language level and motor abilities. EEG recording performed 2 days after the second administration of tocilizumab revealed generalized slow wave activity predominantly localized in temporal regions.	A dramatic cessation of seizures within 24 h;and the consciousness returned to baseline on 7 days after the first administration of tocilizumab; EEG showed no abnormalities after the third administration of tocilizumab
Follow-up			At 1-year follow-up, he has approximately 0-1 seizures per day on 5 ASMs. IQ subsets for processing speed, performance, and verbal IQ are 60, 86, and 81, respectively. Repeat MRI showed right mesial temporal sclerosis in the area of prior T2/FLAIR signal abnormality.	After 1 year, he experienced brief monthly focal seizures, treated with three ASMs, and his EEG showed rare left temporal slow waves. His neuropsychological evaluation is normal, except a small attention deficit.	He suffered seizures once or twice a month. But three months later, he presented a cluster of seizures in one week with more epileptic activity in VEEG and analyzed IL-6 again, which was normal.	At 10 months follow-up, the seizures were controlled for 3 months and then recurred at a rate of 2-3 times/month.IQ was 76.	The convulsive episodes in the child exhibited a reduction compared to previous occurrences, with no evident seizures observed for a maximum duration of approximately 20 days. The level of consciousness remained impaired.	After half a year of follow-up, the patient had no seizures or cognitive impairment.	At the last follow-up on day 160, the child remained seizure-free, and cognitive functions were assessed to be fully Intact.
Adverse reaction			*N*	*N*	*N*	*N*	*N*	*N*	

Y year-old; N/K not known, Y yes, N no, IVIG intravenous immunoglobulin; IVMP intravenous methyl-prednisolone; LEV levetiracetam, PB phenobarbital, VPA valproic acid, LCM lacosamide, TPM topiramate, CBD cannabidiol, LZP lorazepam, CZP clonazepam, PHT phenytoin, ZNS zonisamide, OXC oxcarbazepine, CLB clobazam.

## 4 Discussion

FIRES is a subcategory of NORSE, defined by a preceding febrile infection 2 weeks to 24 hours before refractory status epilepticus (12). It affects previously healthy individuals and presents with abrupt-onset multifocal or generalized seizures rapidly progressing to super-refractory status epilepticus. EEG shows diffuse slowing and/or multifocal epileptiform discharges, while CSF is normal or mildly abnormal and early MRI may be unremarkable. No clear etiology is usually identified, and patients are typically refractory to treatment. Prognosis is poor, with high mortality and frequent progression to chronic drug-resistant epilepsy with neurocognitive impairment (13). Both previously healthy children in this report developed SRSE following a febrile illness. No definite infectious, metabolic, or genetic etiology was identified, and there was no response to multiple ASMs, anesthetic agents, or first-line immunotherapy. Therefore, a diagnosis of FIRES was established.

Accumulating evidence suggests that patients with NORSE/FIRES develop a pronounced proinflammatory milieu, characterized by elevated levels of cytokines and chemokines in both serum and cerebrospinal fluid (CSF), including interleukin-6 (IL-6), tumor necrosis factor-α (TNF-α), CXCL8/interleukin-8 (IL-8), CCL2, macrophage inflammatory protein-1α (MIP-1α), and interleukin-12p70 (IL-12p70). The release of these mediators is thought to depend on the activation of perivascular and glial cells and may contribute to the clinically observed latent period between the initial infectious trigger and the onset of seizures, potentially by promoting sustained neuroinflammation and lowering seizure thresholds (3, 14).In this study, we describe the clinical manifestation of two pediatric FIRES patients treated with tocilizumab. Two cases of children with a history of infection before the onset of epilepsy rapid emergence of status epilepticus after admission, accompanied by fever. Cerebrospinal fluid analysis showed negative results for etiology and immune antibodies, as well as negative findings in metabolic disease screening. First-line immunotherapy was ineffective, while blood IL-6 levels were found to be elevated. Due to limited conditions at the hospital, cerebrospinal fluid testing could not be performed. Given the prominent elevation of IL-6 in the proinflammatory milieu observed in NORSE/FIRES, activation of perivascular and glial cells may drive sustained neuroinflammation and seizure refractoriness, providing a strong mechanistic rationale for IL-6–targeted therapy with tocilizumab to interrupt this inflammatory cascade and potentially improve seizure control. Although peripheral IL-6 levels were not significantly elevated in our two cases, this does not exclude a critical role of IL-6 mediated neuroinflammation in FIRES. Increasing evidence suggests that FIRES is characterized by a dysregulated innate immune response with prominent intrathecal cytokine activation. Previous studies have demonstrated markedly elevated levels of IL-6 and other inflammatory cytokines in CSF, whereas corresponding serum levels may remain normal or only mildly increased, suggesting a compartmentalized inflammatory response within the central nervous system. This discrepancy may be attributed to bloodbutedy barrier dysfunction and localized cytokine production, which limit the reflection of central inflammation in peripheral circulation (15–17). Importantly, therapeutic strategies targeting the IL-6 pathway are not solely dependent on absolute cytokine levels, but rather on the presumed pathogenic role of IL-6 signaling in sustaining neuroinflammation and epileptogenesis. Tocilizumab has shown efficacy in patients with FIRES/NORSE who were refractory to conventional anti-seizure medications and first-line immunotherapies, even in the absence of markedly elevated serum IL-6 levels (6). Consequently, tocilizumab was administered in our cases. Patient 1 consented to use tocilizumab on the 58th day of the disease course, resulting in transient relief of seizures. Throughout the follow-up period, the patient remained unconscious and consistently experienced intermittent seizures, with no observable seizure activity lasting longer than 20 days. Cranial MRI revealed multiple abnormal signals in the brain and cerebral atrophy. Patient 2 received tocilizumab treatment on the 7th day of the disease course. During the six-month follow-up, no seizures were observed, and there was no significant cognitive decline. No abnormal epileptic waves were detected in VEEG and the previously identified abnormal signal on the cranial MRI was effectively resolved.

So far, 7 pediatric patients of FIRES treated with tocilizumab have been reported (7–11). The clinical symptoms of the patients were relatively consistent, characterized by focal seizures and status epilepticus as the primary manifestations. Concurrent fever was observed in most patients, along with a history of infection preceding the onset of seizures. In the early stages, FIRES often presented as misdiagnosed severe infectious encephalitis or autoimmune encephalitis. However, the medical records indicated that the fever symptoms were less severe than those typically seen in severe encephalitis cases, and no obvious psychotic symptoms were observed. Therefore, it is crucial to exercise caution when diagnosing children whose symptoms are not fully explained and especially when treatment fails to yield desired outcomes.

Numerous studies have indicated that FIRES may be associated with an explosive inflammatory response within the central nervous system, particularly the selective up-regulation of IL-6 in cerebrospinal fluid (1, 18, 19). Frequent, repetitive seizures stimulate an inflammatory cascade associated with an increment in IL-6 levels, thereby inducing disruption of the blood-brain barrier and sustaining seizure activity (20). Prompt termination of the neuroinflammatory cascade is crucial for effective treatment. Nevertheless, prior research has demonstrated that high-dose methylprednisolone and immunoglobulin fail to adequately control neuroinflammation, leading researchers to focus on early implementation of second-line immunotherapy (Anakinra or tocilizumab) as the main direction for further investigation (2, 3).

Tocilizumab, an antagonist of the IL-6 receptor, exerts anticonvulsant effects and mitigates neuronal damage by intercepting IL-6 signal transduction. Among the reported cases, serum IL-6 levels were detected in 6 cases, with 3 cases showing a slight increase and 3 cases within the normal range. Only three cases were tested for cerebrospinal fluid IL-6, all of which exhibited significant elevation. The CSF IL-6 level is considered a crucial biomarker for diagnosis and treatment. Notably, no correlation was observed between serum IL-6 levels and those in cerebrospinal fluid. Even if the serum IL-6 level is not elevated, it does not exclude the use of tocilizumab. However, serum IL-6 should not be disregarded as it can serve as a valuable reference when clinical detection of CSF IL-6 level is unfeasible; an increased serum IL-6 level can support the administration of tocilizumab.

Currently, the use of tocilizumab in the treatment of FIRES is limited, and there is no consensus on the optimal timing and dosage. Among the previously reported 4 cases, tocilizumab was administered within 3 weeks after disease onset in 3 cases, with no observed brain atrophy on MRI. In one case, tocilizumab was administered 36 days after disease onset, and the brain MRI revealed evidence of brain atrophy. Patient 2 who was treated with tocilizumab on the 7th day of the disease course demonstrated effective seizure control, complete resolution of intracranial lesions, and no evidence of cognitive impairment during follow-up. We believe that early administration of tocilizumab (within 3 weeks from disease onset) positively impacts the treatment of FIRES, and if initial immunotherapy proves ineffective after 1 week, initiation of tocilizumab therapy can be considered. Up to now, most of the cases can achieve satisfactory results with two doses of tocilizumab, regardless of the single dose of 12mg/kg or 4mg/kg, there are no significant adverse reactions. In the future, large samples and further prospective studies are needed to evaluate the safety, efficacy, single dose and frequency of tocilizumab.

A limitation of this study is the lack of serial inflammatory biomarker data, particularly CSF IL-6, which restricts objective evaluation of treatment response. Nevertheless, given the difficulty of repeated CSF sampling and funding limitations, clinical improvement remains an important surrogate endpoint. The temporal association between tocilizumab treatment and rapid clinical stabilization in our cases provides indirect support for its efficacy. Future studies with dynamic cytokine monitoring are warranted.

## 5 Conclusion

Herein, we present two pediatric patients of FIRES treated with tocilizumab. FIRES is a disorder characterized by previous febrile episodes and subsequent epileptic encephalopathy, primarily affecting the pediatric population and leading to severe neurological outcomes. We propose that CSF IL-6 holds significant diagnostic and therapeutic implications for FIRES, with serum IL-6 serving as a valuable reference marker. Early identification of FIRES and timely administration of tocilizumab has the potential to mitigate disease progression, decrease patient mortality rates, and enhance the quality of survival.

## Data Availability

The original contributions presented in the study are included in the article/supplementary material. Further inquiries can be directed to the corresponding author.

## References

[1] Specchio N Pietrafusa N . New-onset refractory status epilepticus and febrile infection-related epilepsy syndrome. Dev Med Child Neurol. (2020) 62:110–6. doi: 10.1111/dmcn.14553. PMID: 32372459

[2] Sheikh Z Hirsch LJ . A practical approach to in-hospital management of new-onset refractory status epilepticus/febrile infection related epilepsy syndrome. Front Neurol. (2023) 14:1150496. doi: 10.3389/fneur.2023.1150496. PMID: 37251223 PMC10213694

[3] Van Baalen A . Febrile infection-related epilepsy syndrome in childhood: a clinical review and practical approach. Seizure. (2023) 111:215–22. doi: 10.1016/j.seizure.2023.09.008. PMID: 37703593

[4] Pavone P Corsello G Raucci U Lubrano R Parano E Ruggieri M . Febrile infection-related epilepsy syndrome (FIRES): a severe encephalopathy with status epilepticus. Literature review and presentation of two new cases. Ital J Pediatr. (2022) 48:199. doi: 10.1186/s13052-022-01389-1. PMID: 36527084 PMC9756623

[5] Gaspard N Foreman BP Alvarez V Kang CC Probasco JC Jongeling AC . New-onset refractory status epilepticus: etiology, clinical features, and outcome. Neurology. (2015) 85:1604–13. 10.1212/WNL.0000000000001940 PMC4642147 26296517

[6] Jun JS Lee ST Kim R Chu K Lee SK . Tocilizumab treatment for new onset refractory status epilepticus. Ann Neurol. (2018) 84:940–5. doi: 10.1002/ana.25374. PMID: 30408233

[7] Stredny CM Case S Sansevere AJ Son MB Henderson L Gorman MP . Interleukin-6 blockade with tocilizumab in anakinra-refractory febrile infection-related epilepsy syndrome (FIRES). Child Neurol Open. (2020) 7:2329048X20979253. doi: 10.1177/2329048x20979253. PMID: 33403221 PMC7745547

[8] Girardin ML Flamand T Roignot O Warde MTA Mutschler V Voulleminot P . Treatment of new onset refractory status epilepticus/febrile infection-related epilepsy syndrome with tocilizumab in a child and a young adult. Epilepsia. (2023) 64:e87-e92. doi: 10.1111/epi.17591. PMID: 36961094

[9] Cantarín-Extremera V Jiménez-Legido M Duat-Rodríguez A García-Fernández M Ortiz-Cabrera NV Ruiz-Falcó-Rojas ML . Tocilizumab in pediatric refractory status epilepticus and acute epilepsy: experience in two patients. J Neuroimmunol. (2020) 340:577142. doi: 10.1016/j.jneuroim.2019.577142. PMID: 31935626

[10] Wang Y Ma Y Wang Y Zhang J Liu M Li X . A case of febrile infection-related epilepsy syndrome in children successfully treated with tocilizumab and literature review. Chin J Neurol. (2022) 55:1277–85.

[11] Wan L Liu J Liu J Zhu L Wang W Li SW . Favorable outcomes and FDG-PET changes following tocilizumab treatment for febrile infection-related epilepsy syndrome in a child. Int Immunopharmacol. (2025) 146:113872. doi: 10.1016/j.intimp.2024.113872. PMID: 39689594

[12] Hirsch LJ Gaspard N Van Baalen A . Proposed consensus definitions for new-onset refractory status epilepticus (NORSE), febrile infection-related epilepsy syndrome (FIRES), and related conditions. Epilepsia. (2018) 59:739–44. doi: 10.1111/epi.14016. PMID: 29399791

[13] Wickstrom R Taraschenko O Dilena R . International consensus recommendations for management of new onset refractory status epilepticus (NORSE) incl. febrile infection-related epilepsy syndrome (FIRES): statements and supporting evidence. Epilepsia. (2022) 63:2840–64. 10.1111/epi.17397 PMC9828002 35997591

[14] Hanin A Cespedes J Dorgham K . Cytokines in newonset refractory status epilepticus predict outcomes. Ann Neurol. (2023) 94:75–90. doi: 10.1002/ana.26627. PMID: 36871188

[15] Kramer U Chi CS Lin KL Specchio N Sahin M Olson H . Febrile infection-related epilepsy syndrome (FIRES): pathogenesis, treatment, and outcome: a multicenter study on 77 children. Epilepsia. (2011) 52:1956–65. doi: 10.1111/j.1528-1167.2011.03250.x. PMID: 21883180

[16] Van Baalen A Häualer M Boor R Rohr A Sperner J Kurlemann G . Febrile infection-related epilepsy syndrome (FIRES): a nonencephalitic encephalopathy in childhood. Epilepsia. (2010) 51:1323–8. doi: 10.1111/j.1528-1167.2010.02535.x. PMID: 20345937

[17] Sakuma H Tanuma N Kuki I Takahashi Y Shiomi M Hayashi M . Intrathecal overproduction of proinflammatory cytokines and chemokines in febrile infection-related refractory status epilepticus. J Neurol Neurosurg Psychiatry. (2015) 86:820–2. doi: 10.1136/jnnp-2014-309388. PMID: 25398790

[18] Kothur K Bandodkar S Wienholt L Chu S Pope A Gill A . Etiology is the key determinant of neuroinflammation in epilepsy: elevation of cerebrospinal fluid cytokines and chemokines in febrile infection-related epilepsy syndrome and febrile status epilepticus. Epilepsia. (2019) 60:1678–88. doi: 10.1111/epi.16275. PMID: 31283843

[19] Serino D Santarone ME Caputo D Fusco L . Febrile infection-related epilepsy syndrome (FIRES): prevalence, impact and management strategies. Neuropsychiatr Dis Treat. (2019) 15:1897–903. 10.2147/NDT.S177803 PMC6635824 31371963

[20] Vezzani A French J Bartfai T Baram TZ . The role of inflammation in epilepsy. Nat Rev Neurol. (2011) 7:31–40. doi: 10.1038/nrneurol.2010.178. PMID: 21135885 PMC3378051

